# Genetic Markers for Western Corn Rootworm Resistance to Bt Toxin

**DOI:** 10.1534/g3.114.016485

**Published:** 2015-01-07

**Authors:** Lex E. Flagel, Shilpa Swarup, Mao Chen, Christopher Bauer, Humphrey Wanjugi, Matthew Carroll, Patrick Hill, Meghan Tuscan, Raman Bansal, Ronald Flannagan, Thomas L. Clark, Andrew P. Michel, Graham P. Head, Barry S. Goldman

**Affiliations:** * Monsanto Company, Chesterfield, Missouri; † Monsanto Company, Creve Coeur, Missouri; ‡ Department of Entomology, Ohio Agricultural Research and Development Center, Wooster, Ohio

**Keywords:** *Bacillus thuringiensis*, *Diabrotica virgifera virgifera*, genetic marker, insect resistance, western corn rootworm

## Abstract

Western corn rootworm (WCR) is a major maize (*Zea mays* L.) pest leading to annual economic losses of more than 1 billion dollars in the United States. Transgenic maize expressing insecticidal toxins derived from the bacterium *Bacillus thuringiensis* (Bt) are widely used for the management of WCR. However, cultivation of Bt-expressing maize places intense selection pressure on pest populations to evolve resistance. Instances of resistance to Bt toxins have been reported in WCR. Developing genetic markers for resistance will help in characterizing the extent of existing issues, predicting where future field failures may occur, improving insect resistance management strategies, and in designing and sustainably implementing forthcoming WCR control products. Here, we discover and validate genetic markers in WCR that are associated with resistance to the Cry3Bb1 Bt toxin. A field-derived WCR population known to be resistant to the Cry3Bb1 Bt toxin was used to generate a genetic map and to identify a genomic region associated with Cry3Bb1 resistance. Our results indicate that resistance is inherited in a nearly recessive manner and associated with a single autosomal linkage group. Markers tightly linked with resistance were validated using WCR populations collected from Cry3Bb1 maize fields showing significant WCR damage from across the US Corn Belt. Two markers were found to be correlated with both diet (*R*^2^ = 0.14) and plant (*R*^2^ = 0.23) bioassays for resistance. These results will assist in assessing resistance risk for different WCR populations, and can be used to improve insect resistance management strategies.

The western corn rootworm (WCR)—*Diabrotica virgifera virgifera* LeConte (Coleoptera: Chrysomelidae)—is a major maize pest causing more than 1 billion dollars of economic losses annually (Gray *et al.* 2009). In 2003, transgenic maize expressing the Cry3Bb1 insecticidal protein from the bacterium *Bacillus thuringiensis* (Bt maize) was introduced to control WCR (Ward *et al.* 2005). Since then, these Bt maize varieties have been increasingly adopted by US farmers (James 2012), because they offer several benefits, including effective pest management (Hutchison *et al.* 2010), decreased use of chemical insecticides and decreased harm to nontarget organisms (Brookes and Barfoot 2012a), and economic gains for farmers (Brookes and Barfoot 2012b).

The widespread adoption of Bt maize and practices such as continuous maize cultivation create an intense selection pressure on WCR to develop resistance (Cullen *et al.* 2013). In addition, WCR has high levels of genetic diversity (Flagel *et al.* 2014) and has demonstrated the genetic potential to overcome many management tactics, including Bt maize (Gassmann *et al.* 2011), chemical insecticides (Meinke *et al.* 1998; Parimi *et al.* 2006), and crop rotation (Krysan *et al.* 1986). Field-evolved resistance to Bt maize was first reported in Iowa among WCR populations collected in 2009 from fields with a history of continuous cultivation of maize expressing the Cry3Bb1 Bt toxin (Gassmann *et al.* 2011). Subsequent studies performed on populations collected in 2010 confirmed the initial report of resistance (Gassmann *et al.* 2012), and demonstrated that these insects were cross-resistant to the mCry3A toxin (Gassmann *et al.* 2014).

To combat Cry3Bb1 resistance in WCR, insect resistance management (IRM) and integrated pest management (IPM) practices have been implemented (Gould 1998; Petzold-Maxwell *et al.* 2013), with the ultimate goal of slowing the rate at which resistance evolves (Head and Greenplate 2012). IRM strategies are rooted in population genetic theory, and as such require some knowledge of the underlying genetics of resistance. For example, knowing the frequency of the resistance alleles in natural populations can enhance the IRM strategy. In WCR, the genetics of Cry3Bb1 resistance, and therefore the resistance allele frequency, is unknown. However, we can begin to understand the underlying genetics by identifying genetic markers associated with resistance. In addition, genetic markers can be used to predict where field failures may occur and in the design of future products for WCR control.

In this study, we develop a genotyping system and construct a genetic map for WCR, and use these tools to identify and validate genetic markers associated with Cry3Bb1 resistance among field-collected populations. Our results indicate that the inheritance of Cry3Bb1 resistance is associated with a single autosomal linkage group and is nearly completely recessive. We also demonstrate that genetic markers at this locus are predictive of the resistance phenotype across 80 field-collected WCR populations from throughout the US Corn Belt. The impact of this work lies in deciding how and where to implement IRM and IPM practices, which will ultimately increase the sustainability WCR management tactics.

## Materials and Methods

### Preparing WCR mapping families and phenotyping

WCR rearing and selection was conducted on live maize plants under controlled conditions in a growth chamber (25°, 70% relative humidity, 14 hr days, 10 hr nights). We obtained the Hopkinton Cry3Bb1-resistant strain (HopR) (Gassmann *et al.* 2011) from Aaron Gassmann (Iowa State University). A full description of the development of the HopR strain is found in Ingber (2014). The Brookings Cry3Bb1 susceptible strain (BrookS) (Branson 1976) was obtained from the US Department of Agriculture North Central Agricultural Research Laboratory (Brookings, SD). We initiated 40 single-pair crosses between a HopR male and a virgin BrookS female (Supporting Information, Figure S1). The F_1_ generation was reared on seedling mats of germinated non-Bt maize and allowed to randomly sib-mate. F_2_ eggs were reared on germinated seedling mats of Cry3Bb1 expressing maize (treatment) or a genetic near isoline that lacks the Cry3Bb1 transgene (control). F_2_ adult survivors were collected as they emerged from the soil. Animals from all three generations were sexed and stored at −80° before DNA extraction.

### Genotyping and validation

Each focal single-nucleotide polymorphism (SNP) for genotyping by sequencing (GBS) was flanked by a pair of polymerase chain reaction primers (File S1), and after DNA extraction, SNP targets were amplified using the Ion AmpliSeq (Life Technologies) multiplex polymerase chain reaction kit. Amplicons from each animal were uniquely barcoded via adapter ligation, pooled in groups of 96 individuals, and sequenced using an Ion PGM DNA sequencer (Life Technologies). All sequence reads were quality filtered, trimmed of barcodes, and aligned to a library of the expected amplicons using TMAP (TMAP Aligner: https://github.com/iontorrent/TMAP). SNP allele counts and genotype calls for all individuals can be found in File S2.

Two experiments were performed to validate the accuracy of the WCR GBS platform. First 96 individuals were genotyped by GBS in repeat runs generating 50,148 shared genotype calls. A total of 95.6% of shared calls where identical, indicating high technical repeatability. Second, 8−11 individuals were genotyped with 27 cleaved amplified polymorphic sequence markers, as described in Flagel *et al.* (2014). These same individuals and SNP loci also were genotyped with GBS. Among 244 shared genotype calls between cleaved amplified polymorphic sequence and GBS markers, 95.9% were the same, indicating high accuracy.

### Genetic map construction

For each population, we separated markers into three groups: F_1_-informative, F_2_-informative, and sex-linked. Within each population F_1_-informative markers from the F_1_ generation were coded following Wu *et al.* (2002) and assembled into linkage maps using the OneMap package in R (Margarido *et al.* 2007). F_2_-informative markers from each control F_2_ population (treatment F_2_ populations were not used in building the genetic map) were assembled into linkage maps under a standard F_2_ intercross design implemented in OneMap. F_2_-informative sex-linked markers (*i.e.*, parental genotypes X_A_X_A_♀ and X_B_♂) from the control F_2_ population were assembled into linkage groups using a standard F_2_ backcross design implemented in OneMap, with the maternal genotype treated as the recurrent parent and haploid F_2_ males recoded accordingly. All maps were constructed using the Kosambi mapping function and the rapid chain delineation algorithm to order linked markers. We produced six autosomal linkage maps and three X-chromosome linkage maps. A composite linkage map was made for the nine autosomal linkage groups and the X linkage group using MergeMap (Wu *et al.* 2011). This composite map (File S1) was used for all map-based analyses.

### Population collection and bioassays

WCR adults were collected from 80 locations in response to grower reports of injury to Cry3Bb1-expressing maize from June-September in 2013. Damaged fields were evaluated for the presence of the Cry3Bb1 protein using lateral flow immunoassay strips (Envirologix). A nodal injury score (NIS), ranging from 0 to 3 was calculated for 10−15 plants to assess WCR damage. Adult WCR were collected from SmartStax-RIB Complete fields if at least 50% of the plants evaluated had a NIS ≥0.75 or VT Triple Pro fields with NIS ≥1. Populations were sent to Custom Bio Products (Maxwell, IA) or to the Monsanto Insectary (Waterman, IL) for rearing. Offspring from each population were used to perform plant and diet bioassays.

Plant bioassays were conducted as described earlier (Clark and Hibbard 2004), using 10 replicates of 30 neonate larvae from each population on Cry3Bb1 expressing maize, and 10 additional replicates on a genetic near isoline lacking the Cry3Bb1 transgene. Plants were grown in a greenhouse in containers with 50% silty loam and 50% Metro 200 soil and watered as needed. The greenhouse was maintained at 25°, 75% relative humidity, and 14-hr days, 10-hr nights. We confirmed the presence of Cry3Bb1 in Bt maize plants and absence in non-Bt maize plants with assay strips as described previously. At V4−V5 growth stage, plants were infested with 30 neonate WCR larvae aged less than 24 hr. Approximately 10 days after infestation, we collected larvae for 3 d using a Berlese/Tullgren funnel method. Larvae emerging from the funnel were counted. The plant bioassay resistance score was calculated by taking the average number of survivors from the Bt plants divided by the average number of survivors from non-Bt plants.

To estimate the repeatability of replicate plant bioassays, we fitted a Poisson regression using the following model: ln(*trt_ij_*) = *pop_j_* + ln(*ctl_ij_*), where *trt* and *ctl* are the adult survivor counts among 30 insects assigned to both the Cry3Bb1 treatment and near isoline control, respectively, *pop* is the population variable, and *i* indexes the bioassay replicate within population *j*. The *R*^2^ for the population term in the fitted model was estimating using McFadden’s method.

Diet bioassays were performed at Custom Bio Products in standard 96-well tissue culture trays supplied by Monsanto Company (Chesterfield, MO). Individual neonate larvae (<24 hr old) were randomly selected and placed in the wells. Each population was assigned to 12 wells per plate and this was replicated among six plates. WCR larvae were treated with the Cry3Bb1 protein in buffer (170.8 µg of protein per cm^2^ of diet surface) or a control buffer lacking Cry3Bb1. A total of 20 µL of either solution was overlaid on each well and allowed to air dry prior to larval infestation. Mortality was recorded after five days of incubation in the dark at 27° and 40–50% relative humidity. The diet bioassay resistance score was calculated by taking the average number of survivors from the Cry3Bb1 treatment divided by the average number of survivors from the buffer control. For plant and diet bioassays, a score of 1 indicates the greatest resistance, whereas 0 indicates the greatest susceptibility to Cry3Bb1.

### Genotyping field populations

Six GBS markers (CRW663, CRW996, CRW1622, CRW1683, CRW1862, and CRW1878; File S1) from linkage group 8 (LG8) were selected for characterization among the field-collected populations. Each marker was converted to a TaqMan (Life Technologies) SNP genotyping assay. To determine the accuracy of the TaqMan markers, we used them to genotype 62 individuals that had been previously genotyped using GBS. For all six markers, there was perfect agreement between the TaqMan and GBS genotypes. WCR DNA was extracted using a protocol based on Dellaporta *et al.* (1983) and TaqMan assays were performed following the manufacturer’s protocol. From each population allele frequency was estimated from 48 individuals.

## Results

### WCR genotyping platform and mapping population

To develop a genotyping platform for WCR we used SNPs identified by Flagel *et al.* (2014). We selected 1150 biallelic SNPs with a high average expected heterozygosity (*H_e_* > 0.25) among 26 populations. For each SNP a GBS assay was developed (File S1). Twenty-seven GBS assays were selected for independent validation, and the observed accuracy was 95.9% (see the section *Materials and Methods*).

A WCR population with field-evolved resistance to Cry3Bb1 was collected near Hopkinton, Iowa, in 2009 by Gassmann *et al.* (2011). To accelerate the generation time, the diapausing Hopkinton population was introgressed with a nondiapausing Cry3Bb1 susceptible population (BrookS) (Ingber 2014) maintained by the USDA in Brookings, SD. Individuals from this mixed population were kindly shared by Dr. Aaron Gassmann and were selected in the laboratory for Cry3Bb1 resistance and nondiapausing development for an additional five generations to create a nondiapausing, Cry3Bb1-resistant population (HopR). Initial test-crosses between BrookS and HopR strains indicated that Cry3Bb1 resistance had nearly recessive inheritance and was not sex-linked. With this knowledge, we initiated 40 single-pair crosses between a HopR male and a virgin BrookS female. Each surviving family was reared to the F_2_ generation by allowing random mating among F_1_ siblings (Figure S1). Three families (#11, #24, and #37) produced sufficient F_2_ offspring and were selected for further analysis. For each family all surviving individuals (parents, F_1_s, and F_2_s) were genotyped at 1150 SNP markers, resulting in approximately 1.4 million genotype calls (File S2).

### Constructing a WCR genetic map

Common schemes for building a genetic map involve mating parents to generate a F_2_ intercross, F_2_ backcross, or recombinant inbred line mapping population. These methods require inbred parental lines, but inbreeding depression in WCR prevented their development. Instead, we developed a scheme that made use of outbred parental crosses. For each of the three BrookS × HopR families, we used the F_1_ offspring to create a genetic map among F_1_-informative markers, and the F_2_ offspring to create another map using F_2_-informative markers (Figure S1). Sex-linked markers were identified and used to create an X chromosome map (see the section *Materials and Methods*). All sex-linked markers showed segregation consistent with XO sex-determination, which is supported by the cytogenetic literature on WCR and other species in the genus *Diabrotica* (Ennis 1972; Stevens 1908).

The aforementioned mapping process was carried out independently for all three mapping families. The F_1_-informative autosomal maps contained the most informative markers (*N* = 348-438) and shared nine autosomal linkage groups, consistent with earlier cytogenetic analysis in WCR (Ennis 1972). All markers shared between different mapping populations were assigned to congruent linkage groups. The component maps (autosomal F_1_, autosomal F_2_, and X chromosome maps) from all three families were integrated into a single composite map (see the section *Materials and Methods*). This composite map contains 10 linkage groups, 770 markers, and spans 2325 cM with 3.1 cM between markers on average (File S1). We anticipate that this genetic map may have few markers in regions of the genome tightly linked to the nondiapause trait because the HopR population was recently introgressed with the BrookS population to confer this phenotype.

### Mapping Cry3Bb1 resistance

F_2_ eggs from each BrookS X HopR family were partitioned randomly into treatment (reared on Cry3Bb1-expressing maize) and control groups (reared on a genetic near isoline maize that lacks the Cry3Bb1 transgene) and placed accordingly on germinated seedling mats. Treatment and control survivors were collected and genotyped. To identify markers associated with Cry3Bb1 resistance we performed χ^2^ tests of independence between genotypic counts among treatment and control F_2_ survivors. In total, 1497 χ^2^ tests were performed, each with 2 degrees of freedom, resulting in a Bonferroni significance threshold of χ^2^ ≥ 20.6.

**Figure 1:**
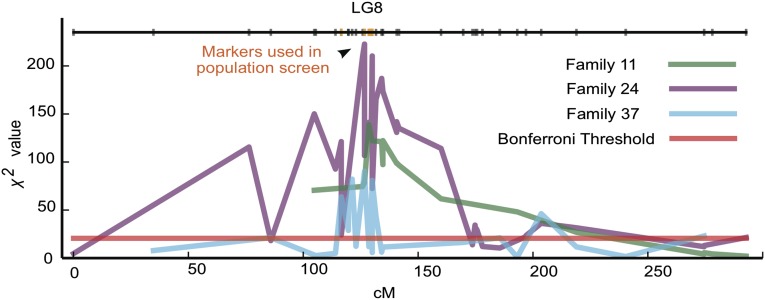
Cry3Bb1 resistance locus on LG8. The only significant response to Cry3Bb1 selection is shown here. The χ^2^ value is derived from genotypic counts among treatment and control F_2_ populations for Family 11 (green), Family 24 (purple), and Family 37 (cyan). The Bonferroni threshold is indicated as a red line. A schematic representation of the LG8 composite linkage map is given at the top of the plot. Six candidate markers used in population screens are shown in orange. LG8, linkage group 8.

In our segregating BrookS X HopR families, a single locus on LG8 appeared to be the only locus with a significant response to Cry3Bb1 selection (Figure 1 and Figure S2). Among all three families, the maximum χ^2^ value for markers in this region was >90, which is equivalent to a *P*-value < 2.9 × 10^−20^. Although in our three mapping families we find strong evidence in favor of one locus, it is possible that other resistance loci were lost during population construction.

To determine whether other forces could be responsible for the large genotypic distortion on LG8, we examined the impact of segregation distortion among the F_2_ control populations. This is particularly powerful because the F_2_ control populations experienced all the forces that could impact genotype frequencies across three generations except Cry3Bb1 selection. Genome-wide there are no significant signatures of segregation distortion among the F_2_ control populations and the observed changes in marker frequencies are consistent with the small expected deviations caused by random genetic drift (Figure S3). This leaves Cry3Bb1 selection as the lone explanatory agent for the sizeable genotypic distortion found between treatment and control populations on LG8.

### Inheritance and selective advantage of the Cry3Bb1 resistance locus

Among all three families, the portion of LG8 that responds most strongly to Cry3Bb1 selection falls between approximately 115 and 135 cM. There are 39 markers in this interval (File S1), and among these markers the inheritance of resistance appears to be predominantly recessive. For example, most F_2_ survivors of Cry3Bb1 treatment are homozygous for the resistant HopR paternal genotype, with a few heterozygous survivors (Figure S4). In addition, in this interval there were almost no survivors of the Cry3Bb1 treatment who were homozygous for the susceptible BrookS maternal genotype. In contrast, the observed genotype counts on LG8 among the control F_2_ populations were consistent with the expected 1:2:1 ratio (Figure S4).

Because F_2_ eggs were assigned randomly to experimental treatments, we expect the initial genotypic frequencies of both groups to differ only by random sampling error. Using a Monte Carlo simulation conditioned on the F_2_ sample sizes (Figure S1), we estimated that the expected sampling error between treatment and control F_2_ genotype frequencies has a median absolute deviation ≤ 4.6% for all three families (File S3). Because this sampling error expected for initial genotype frequencies is small, we can use the final genotype frequencies among treatment and control F_2_s to estimate the relative fitness advantage conferred by the LG8 Cry3Bb1 resistance locus among WCR reared on Cry3Bb1-expressing maize. For these calculations, we selected the F_2_-informative marker with the greatest χ^2^ value between treatment and control groups for each family. Table 1 shows the relative fitness of the three genotypic classes (BrookS/BrookS, BrookS/HopR, and HopR/HopR) among the Cry3Bb1-treated F_2_s. When exposed to Cry3Bb1, the average fitness of the BrookS/BrookS genotype among all three families is nearly zero, whereas the BrookS/HopR heterozygote has on average only 12% of the fitness of HopR/HopR homozygote. Because the fitness of BrookS/BrookS homozygote is nearly zero, the estimated degree of dominance of the susceptible BrookS allele over the HopR resistance allele is equal to one minus the average fitness of the BrookS/HopR heterozygote (*i.e.*, 0.88). Thus the susceptible BrookS allele is primarily dominant, and correspondingly the HopR resistance allele is nearly recessive.

**Table 1:** Marker name, position on LG8, χ^2^ value, parental genotypes, and estimated relative fitness of the three genotypic classes among F_2_ survivors of Cry3Bb1 treatment are given for each family

Family	Marker	Position, cM	χ^2^ Value	Resistant HopR Genotype	Susceptible BrookS Genotype	*w*_SS_	*w_RS_*	*w*_RR_
#11	CRW1878	128.6	138.9	GG	CC	0	0.036	1
#24	CRW1059	126.7	222.7	GG	CC	0	0.024	1
#37	CRW394	116.6	63.8	AA	GG	0.01	0.31	1

*w*_SS_, *w*_RS_, and *w*_RR_ represent, respectively, the relative fitness among Cry3Bb1-treated F_2_s for the BrookS/BrookS, BrookS/HopR, and HopR/HopR genotypes. For each family, the most significant F_2_-informative marker is given. The χ^2^ values given in the table translate to *P*-values of 6.9 × 10^−31^, 4.4 × 10^−49^, and 1.4 × 10^−14^, respectively, for families #11, #24, and #37.

Family 37 contained more heterozygous survivors of Cry3Bb1 selection (Figure S4), and the resistance locus was considerably less recessive than in the other families (Table 1). There are at least three possible explanations for this observation. The environmental conditions could have been different for this family (hypothesis 1). For example, the Cry3Bb1 dosage delivered to Family 37 could have been lower, in turn increasing the effective dominance of the resistance allele. Or, because the parents were not genetically identical, the HopR parent for Family 37 could have contributed a less recessive Cry3Bb1 resistance locus at LG8 (hypothesis 2). Or finally, the HopR parent could have contributed additional unlinked resistance loci that act epistatically with the locus on LG8 (hypothesis 3). We were able to test this third hypothesis in Family 37 by focusing on F_2_ survivors of the Cry3Bb1 treatment that were heterozygous for the founding parent haplotypes in the LG8 resistance interval (*N* = 68). Conditioning on these individuals, we held the effect of the LG8 locus constant, allowing us to compare their genotype frequencies to the control F_2_ population across the rest of the genome. Although we had ample statistical power, we found no evidence for additional resistance loci that work in conjunction with the one on LG8 (Figure S5). This confirms that the locus on LG8 is solely responsible for Cry3Bb1 resistance in Family 37. However, at the present time we cannot determine whether environmental variation (hypothesis 1) or genetic variation at the LG8 resistance locus (hypothesis 2) is responsible for the added dominance observed in Family 37.

### Integrating resistance mapping with field populations

**Figure 2:**
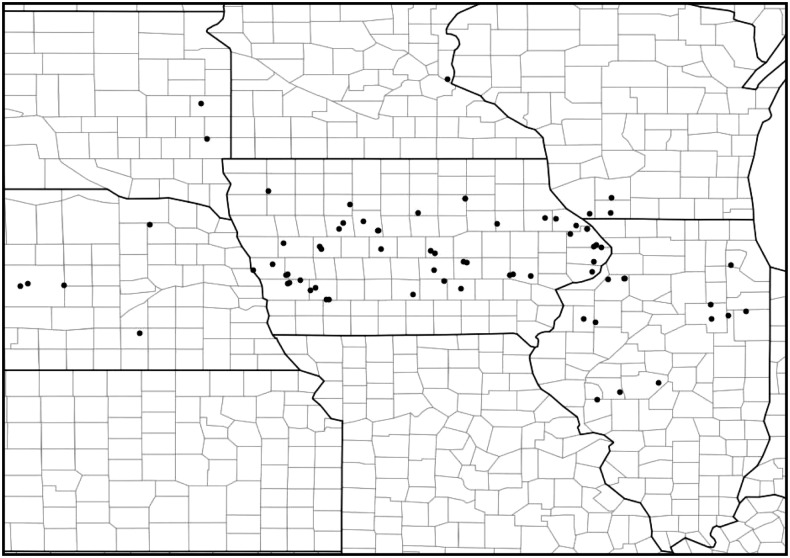
WCR problem field locations across the US Corn Belt. WCR were collected from the 80 locations indicated. Sections within a state are individual counties. WCR, Western corn rootworm.

**Figure 3:**
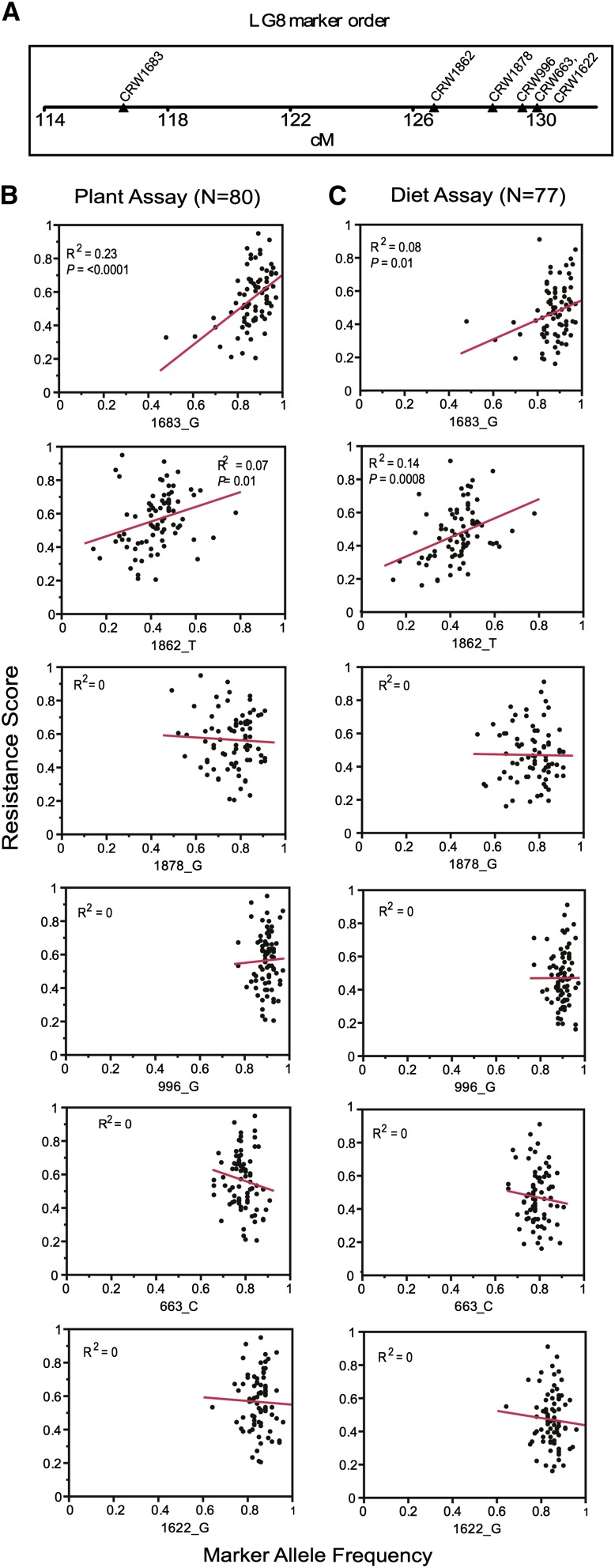
Predictability of six candidate markers. The map positions of the six markers tightly linked to the LG8 resistance locus are shown (A). Regression analysis of resistance scores (y-axis) and the allele frequency of the SNP allele found in resistant HopR parent (x-axis) are shown for each marker for plant (B) and diet (C) bioassays. The marker name and single-nucleotide polymorphism allele are joined by an underscore and listed under each plot. A resistance score of 1 indicates complete resistance, whereas 0 indicates complete susceptibility to Cry3Bb1. LG8, linkage group 8.

To test whether the LG8 resistance locus is common among problem field populations throughout the US Corn Belt, we genotyped approximately 80 populations (Figure 2) for six markers tightly linked to the LG8 resistance locus (Figure 1). In addition, these populations were also phenotypically scored with the use of both plant and diet bioassays to estimate their resistance to Cry3Bb1. Least-squares linear regression analysis on resistance scores from bioassays and the frequency of the SNP allele associated with the resistant HopR parent was performed to test the predictive value for all six markers (Figure 3). Two markers—CRW1683 and CRW1862—were found to be significantly positively correlated with resistance (*P* < 0.05) in plant and diet bioassays. CRW1683 and CRW1862 show modest explanatory power to predict resistance scores (plant bioassays *R*^2^ = 0.23 and 0.07, respectively). After fitting a Poisson regression (see the section *Materials and Methods*) to repeated plant bioassay measures among all populations, we found that the *R*^2^ for the population term in the model was 0.33. This indicates considerable variation among replicated plant bioassays for many populations, which translates into uncertainty in the estimated resistance score. This variation limits the explanatory power of our genetic markers, leaving, for example, only modest room for improvement of the predictive value for marker CRW1683 in the plant bioassays.

## Discussion

WCR is among the most damaging maize pests in the United States but can be managed effectively with plant-expressed Bt toxins such as Cry3Bb1. Recent studies, however, have reported resistance to Cry3Bb1 in a number of WCR populations (Gassmann *et al.* 2011, 2012, 2014). To ensure the long-term viability of Cry3Bb1, IRM and IPM practices have been implemented, but these management efforts can be enhanced with a more specific knowledge of the distribution of the resistance alleles. With genetic markers for resistance, we can estimate the resistance marker frequency in the field, assess resistance risk for different locations, decide how and where to implement IRM practices, and determine the efficacy of these practices.

The identification of genetic markers for Bt toxin resistance in WCR is challenging because 1) this species lacks a complete genome sequence, genetic linkage map, and collections of inbred lines; 2) there is a limited understanding of the mode of action of Bt toxins in WCR and thus no ready pool of candidate genes for Cry3Bb1 resistance; and 3) there is difficulty in measuring resistance at the individual insect level using a robust assay. Our work is novel because we overcome these constraints to produce a genetic linkage map and to identify a locus associated with Bt-resistance. In so doing, we show that resistance is nearly recessive and localized to a single region on LG8. Moreover, we identify two genetic markers from this locus that are predictive of resistance among field-collected populations. This demonstrates that the LG8 resistance locus is likely shared across multiple resistant populations from a broad geography.

Our work lays a foundation for future efforts to measure the frequency of Cry3Bb1 resistance alleles among WCR populations. This will in turn help in the progression from preventive resistance management in WCR, which implements IRM practices without assessing resistant allele frequency, to adaptive resistance management, which uses estimates of resistance allele frequencies to make management decisions.

Genetic markers for Bt toxin resistance have been developed in lepidopteran species (Heckel *et al.* 1999; Baxter *et al.* 2005, 2011; Park *et al.* 2014) and in some cases the causal loci have been identified (Gahan *et al.* 2001, 2010; Atsumi *et al.* 2012). To our knowledge, our work represents the first genetic mapping study in WCR and the first identification of Bt toxin resistance markers in any coleopteran species. These findings provide the foundation for further characterization of this trait in WCR and more broadly in coleopteran insects, where little is known about the mode of action of Bt toxins. Moreover, the SNP markers and genetic map developed for these purposes can be used for trait mapping in other WCR populations.

Of the six SNP markers tested in field populations, the alleles associated with the resistant HopR parent often were found at a high frequency in problem field populations (Figure 3). This observation does not necessarily mean that the resistance allele itself is at a high frequency in these populations. Indeed, there are populations with a high frequency of a resistance-associated SNP allele that are nonetheless sensitive to Cry3Bb1 (Figure 3). This suggests that there could be high linkage disequilibrium between the resistance locus and the resistance-associated SNP alleles in the mapping population (HopR), but not in all populations, a common observation among trait and association mapping studies (Lander and Schork 1994). Despite the potential for imperfect linkage disequilibrium between SNP markers and resistance alleles, the significant positive correlation between marker and phenotype (Figure 3) indicates that these SNPs are valuable for predicting resistance risk.

The region linked to the Cry3Bb1 resistance (115−135 cM on LG8) contains 39 SNP markers. Each SNP marker was discovered within a transcript (Flagel *et al.* 2014), which presents the opportunity to explore the functional annotations of these genes (File S1). Among the 39 genes, we found a match to a putative ABC transporter (marker CRW424 at 119.6 cM), a gene family that is frequently associated with Bt toxin resistance in Lepidoptera (Gahan *et al.* 2010; Park *et al.* 2014). The WCR ABC transporter appears to belong to the ABC*B* subfamily (Figure S6), not the ABC*C* subfamily associated with lepidopteran resistance (Gahan *et al.* 2010; Park *et al.* 2014). Another gene of interest is a Cytochrome P450 that contains marker CRW918 (at 123.0 cM). Cytochrome P450s have not been implicated in Bt resistance in insects, but they have been found to confer resistance to other insecticidal toxins (Scott 1999).

The causal locus need not be among the 39 genes mentioned previously. These genes could simply be tightly linked to the causal locus. Further genetic dissection of this locus will be needed to identify the causal locus. The field population screens offer some clues about where additional research should be focused. The two most predictive markers in field populations (CRW1683 and CRW1862) fall near the center and left side of the resistance locus (Figure 3), making it less likely that the causal alleles fall at a position greater than about 128 cM on the genetic map. One path forward may be to saturate the resistance locus with new genetic markers and fine map resistance in a large segregating population.

## Supplementary Material

Supporting Information
