# Supplementary material for: Genetic Markers for Western Corn Rootworm Resistance to Bt Toxin
Source: G3 (Bethesda). 2015 Jan 7;5(3):399–405. doi: 10.1534/g3.114.016485 (PMC4349093; doi:10.1534/g3.114.016485)
Supplement: Supporting Information [file supp_5_3_399__index.html]

Genetic Markers for Western Corn Rootworm Resistance to Bt Toxin — Supporting Information 

# Genetic Markers for Western Corn Rootworm Resistance to Bt Toxin

## Supporting Information for Flagel *et al.*, 2015

**Files in this Data Supplement:**

- Supporting Information - Figures S1-S6 and Files S1-S3 (PDF, 598 KB)
- Figure S1 - Single-pair mating and mapping scheme used for all three Cry3Bb1 resistance mapping families. (PDF, 155 KB)
- Figure S2 - Difference in genotype frequencies between treatment and control F2 populations for all 10 linkage groups as measured by the chi-squared statistic. (PDF, 204 KB)
- Figure S3 - Tests for segregation distortion among mapping families. (PDF, 226 KB)
- Figure S4 - Resistant parent haplotype enrichment among Cry3Bb1 treated F2s. (PDF, 207 KB)
- Figure S5 - To search for loci unlinked to the LG8 resistance locus that may influence resistance we focused on F2 survivors of the Cry3Bb1 treatmeant that where heterozygous in the resistance interval on LG8. (PDF, 200 KB)
- Figure S6 - Neighbor joining phylogeny of ABC transporter protein sequences from *Tribolium castaneum* (black) and a WCR homolog (red). (PDF, 194 KB)
- File S3 - Python simulation code. (PDF, 113 KB)
- File S1 - Information about the genotyping system and genetic map for WCR. Each genotyping marker is listed along with its position on the genetic map, PCR primer pairs used to amplify it from WCR DNA, and the amplicon these primers produce with the focal SNP position in brackets. (.xlsx, 172 KB)
- File S2 - Comma separated values (.csv) file compressed with zip that contains genotype information for WCR used in the mapping study. Each line of the file gives information for a single marker in one individual. This information includes the marker ("marker") and individual name ("indv"), the family ("family") and generation ("generation") this individual belongs to, the sex ("sex") of the individual and the phenotypic treatment ("pheno", which only applies to the F2 generation and can be CRY3BB1 (i.e. treatment) or ISOLINE (i.e. control)), and finally the inferred genotype ("geno"), and the SNP information from which this genotype was inferred (bases observed ("SNP1\_call" and "SNP2\_call") and their counts ("SNP1\_coverage" and "SNP2\_coverage")). (.zip, 6 MB)
