# Supplementary material for: Genetic Markers for Western Corn Rootworm Resistance to Bt Toxin
Source: G3 (Bethesda). 2015 Jan 7;5(3):399–405. doi: 10.1534/g3.114.016485 (PMC4349093; doi:10.1534/g3.114.016485)
Supplement: Supporting Information [file supp_g3.114.016485_016485SI.pdf]

G3 · Genes | Genomes | Genetics

Genetic Markers for Western Corn Rootworm Resistance to Bt Toxin

Lex E. Flagel[*, 1], Shilpa Swarup[†, 1], Mao Chen[*], Christopher Bauer[*], Humphrey Wanjugi[*], Matthew Carroll[†], Patrick Hill[*], Meghan Tuscan[*], Raman Bansal[‡], Ronald Flannagan[*], Thomas L. Clark[*], Andrew P. Michel[‡], Graham P. Head[†, 2], Barry S. Goldman[*, 2]

[*] Monsanto Company, 700 Chesterfield Parkway W, Chesterfield, MO, 63017, USA

[†] Monsanto Company, 800 N Lindbergh Blvd, Creve Coeur, MO, 63141, USA

[‡] Department of Entomology, Ohio Agricultural Research and Development Center, 1680 Madison Ave., Wooster, OH 44691, USA

[1] These authors contributed equally

[2] To whom correspondence should be addressed: graham.p.head@monsanto.com; barry.s.goldman@monsanto.com

Conflict of interest statement: L.E.F., S.S., M. Chen, C.B., H.W., M. Carroll, P.H., M.T., R.F., T.L.C., G.P.H., and B.S.G. are employed by Monsanto Company, which manufactures maize containing the Cry3Bb1 protein. Some authors are also shareholders in Monsanto Company, though individually or collectively in no way represent any controlling interest in the affairs of Monsanto Company. Results described in the manuscript are the product of work financed by Monsanto Company and the publication fees will be paid by Monsanto Company. The authors are not known to have any financial competing interests in addition to those listed above.

DOI: 10.1534/g3.114.016485

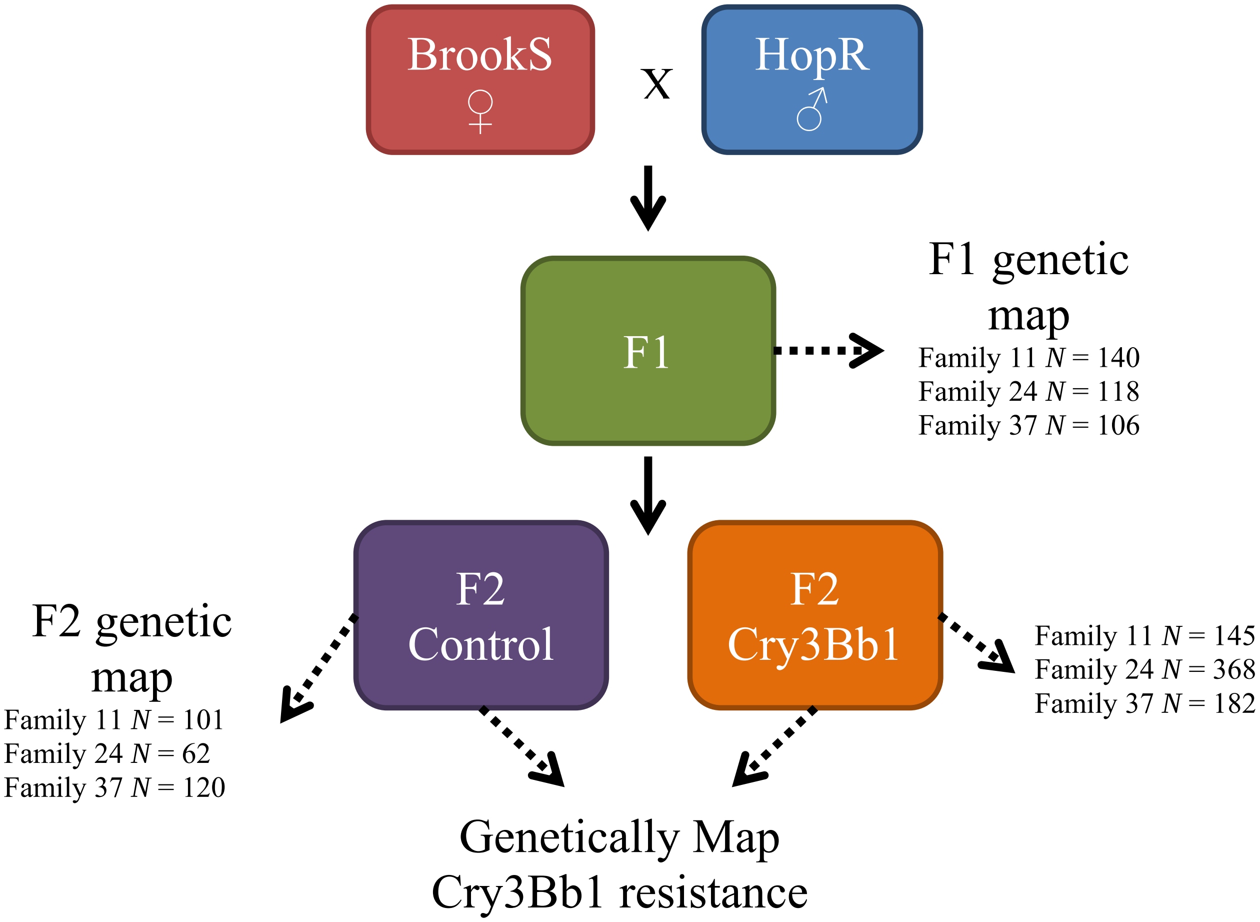

Figure S1 Single-pair mating and mapping scheme used for all three Cry3Bb1 resistance mapping families.

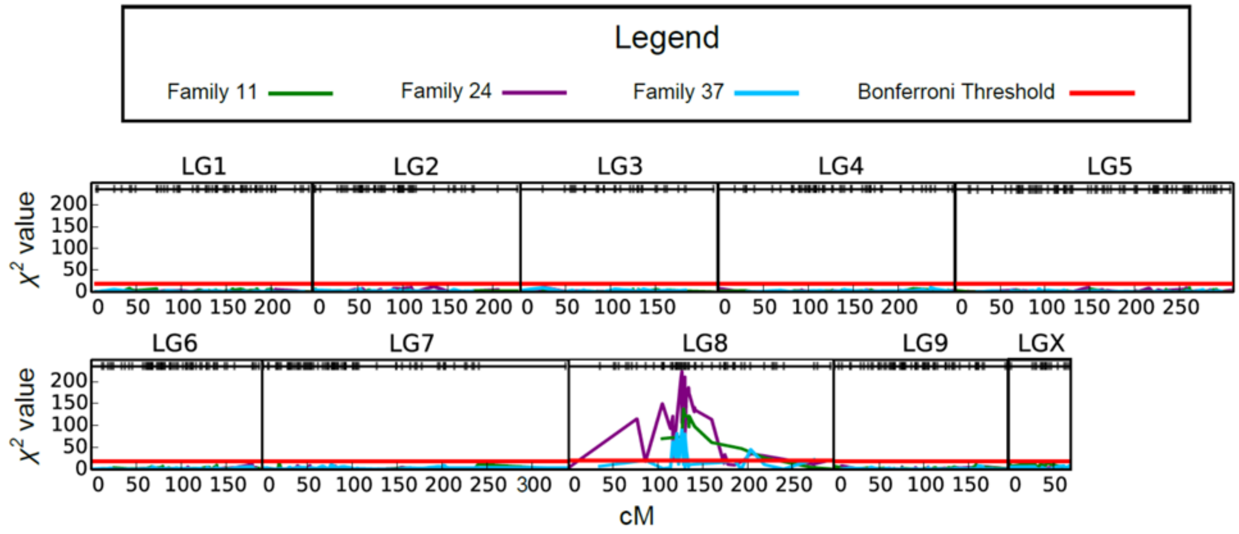

Figure S2 Difference in genotype frequencies between treatment and control F_2 populations for all 10 linkage groups as measured by the chi-squared statistic. The plot below gives a chi-squared test of independence between genotypic counts among treatment and control F_2 survivors for all three families. In total 1,497 tests were performed, each with 2 degrees of freedom, resulting in a Bonferroni significance threshold of $\chi^2 \geq 20.6$. The genotyped marker positions are given at the top of each linkage group panel.

A.

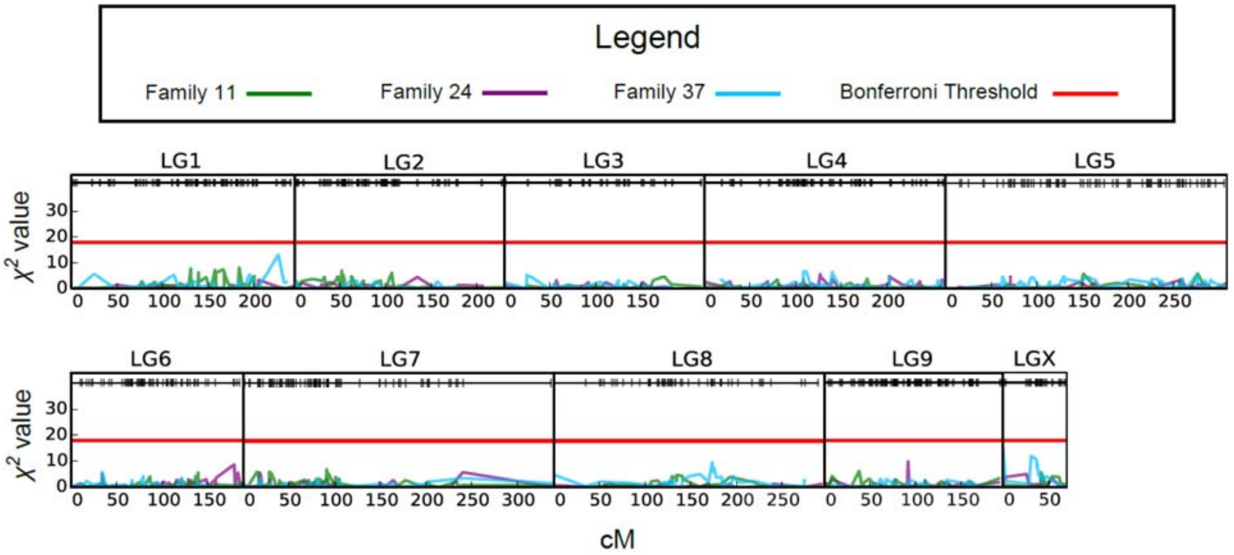

B.

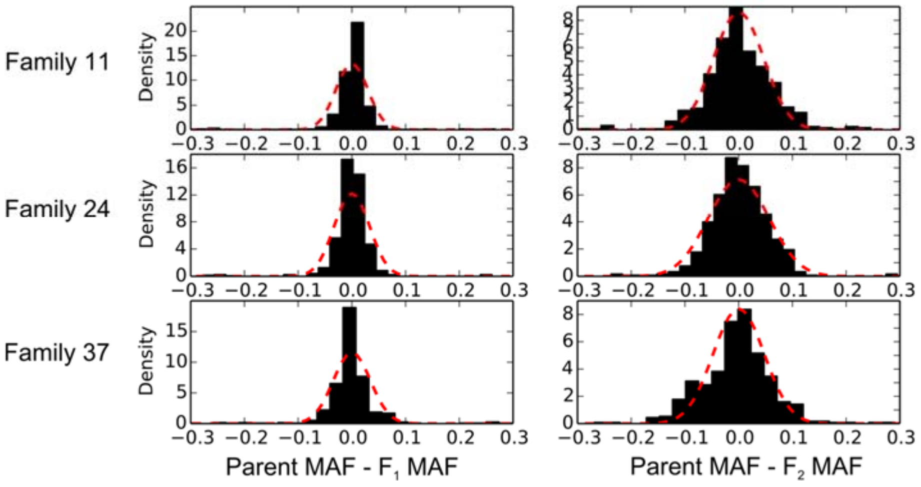

Figure S3 Tests for segregation distortion among mapping families. **(A)** The genomic context of allele frequency segregation distortion between F_1s and the control F_2s across the 10 linkage groups as measured by the chi-squared statistic. 1,713 tests were run, each with 1 degree of freedom, resulting in a Bonferroni significance threshold of $\chi^2 \geq 17.5$. No markers show significant segregation distortion at this threshold. The genotyped marker positions are given at the top of each linkage group panel.

(B) Histograms of the deviation of the parental minor allele frequency (MAF) and the frequency of that same allele in the F_1 and control F_2 generations (black bars) for each of the three families and theoretical expectations based on random genetic drift (red dashed line). Allele frequency deviations are nearly symmetric and follow a distribution consistent with the expectation from random genetic drift.

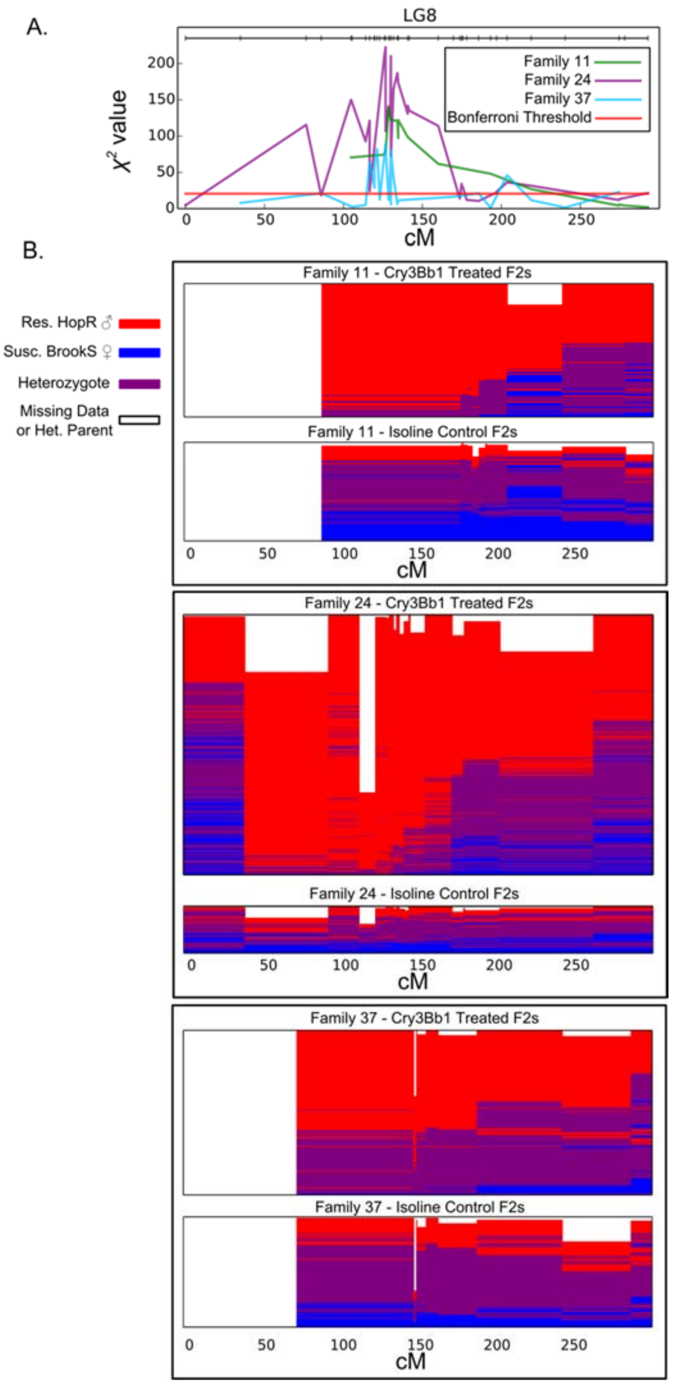

Figure S4 Resistant parent haplotype enrichment among Cry3Bb1 treated F_2s. **(A)** Map of resistance locus on LG8. **(B)** Enrichment of haplotpes contributed by Cry3Bb1 resistant parent in the resistance region on LG8 among F_2 survivors of Cry3Bb1. Each horizontal bar respresent the genotype of a single F_2 individual. Only F_2-informative markers are plotted to ensure correct haplotype phasing. Cry3Bb1 selected F_2s show enrichment for the resistant parent halpotype (red), while all control F2s segregate near the expected 1:2:1 ratio among the resistant parent (red), heterozygote (purple), and susceptible parent (blue) genotypes. Data are missing for the left portion of Family 11 and 37 because the mapping parents lacked F_2-informative markers in this region.

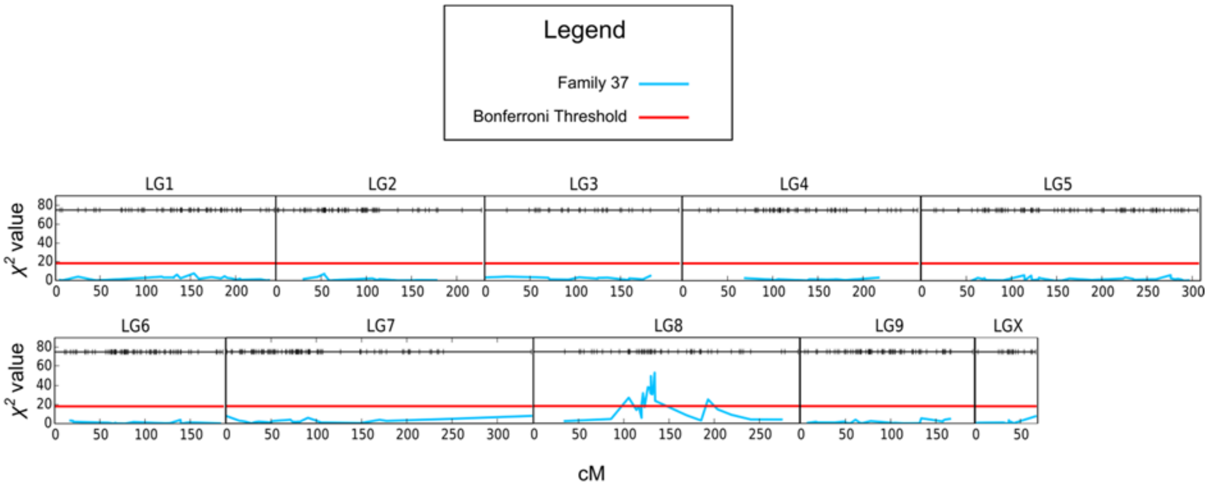

Figure S5 To search for loci unlinked to the LG8 resistance locus that may influence resistance we focused on F_2 survivors of the Cry3Bb1 treatmeant that where heterozygous in the resistance interval on LG8. Conditioning on these individuals as the treatment population, the plot below gives the difference in genotype frequencies between treatment and control F_2 populations for all 10 linkage groups as measured by the chi-squared statistic. In total, 454 tests were performed, each with 2 degrees of freedom, resulting in a Bonferroni significance threshold of $\chi^2 \geq 18.2$. Only the LG8 resistance region is significant (due to the fact that the treatment population was completely heterozygous, while the control population contained resistant parent, heterozygote, and suscpetible parent genotypes). The genotyped marker positions are given at the top of each linkage group panel.

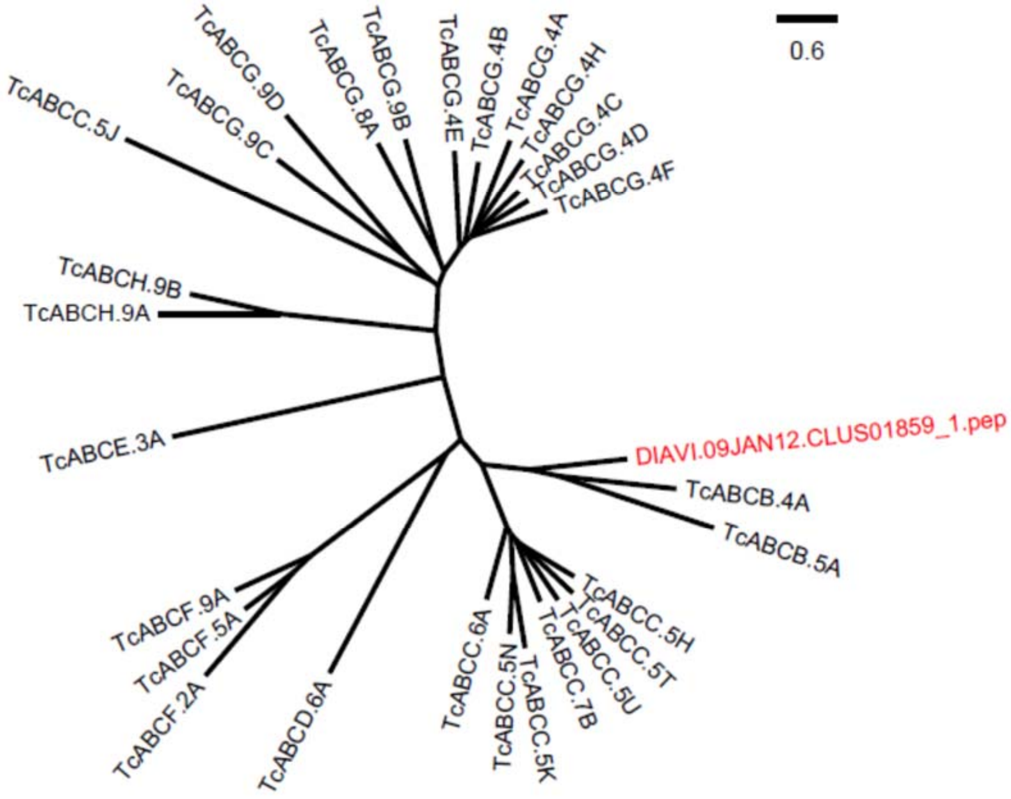

Figure S6 Neighbor joining phylogeny of ABC transporter protein sequences from *Tribolium castaneum* (black) and a WCR homolog (red). The WCR gene contains the CRW424 SNP marker linked to the LG8 resistance locus. The WCR homolog appears to be a member of the ABCB clade. The *T. castaneum* genes were annotated by Broehan *et al.*, 2013 (BMC Genomics 14:6). A substitution scale bar is given in the upper right corner.

Files S1-S2

Available for download at http://www.g3journal.org/lookup/suppl/doi:10.1534/g3.114.016485/-/DC1

File S1 contains information about the genotyping system and genetic map for WCR. Each genotyping marker is listed along with its position on the genetic map, PCR primer pairs used to amplify it from WCR DNA, and the amplicon these primers produce with the focal SNP position in brackets.

File S2 is a comma separated values (.csv) file compressed with zip that contains genotype information for WCR used in the mapping study. Each line of the file gives information for a single marker in one individual. This information includes the marker ("marker") and individual name ("indv"), the family ("family") and generation ("generation") this individual belongs to, the sex ("sex") of the individual and the phenotypic treatment ("pheno", which only applies to the F2 generation and can be CRY3BB1 (i.e. treatment) or ISOLINE (i.e. control)), and finally the inferred genotype ("geno"), and the SNP information from which this genotype was inferred (bases observed ("SNP1_call" and "SNP2_call") and their counts ("SNP1_coverage" and "SNP2_coverage")).

File S3

Python simulation code

```
#Lex Flagel, Monsanto Co.
#10/29/14
#Estimating genotypic sampling error between treatment and control population using
#Monte Carlo
#
#Copyright (c) 2014 Monsanto Co.
#Permission is hereby granted, free of charge, to any person obtaining a copy of this
#software and associated documentation files (the "Software"), to deal in the Software
#without restriction, including without limitation the rights to use, copy, modify,
#merge, publish, distribute, sublicense, and/or sell copies of the Software, and to
#permit persons to whom the Software is furnished to do so, subject to the following
#conditions:
#
#The above copyright notice and this permission notice shall be included in all copies
#or substantial portions of the Software.
#
#THE SOFTWARE IS PROVIDED "AS IS", WITHOUT WARRANTY OF ANY KIND, EXPRESS OR IMPLIED,
#INCLUDING BUT NOT LIMITED TO THE WARRANTIES OF MERCHANTABILITY, FITNESS FOR A
#PARTICULAR PURPOSE AND NONINFRINGEMENT. IN NO EVENT SHALL THE AUTHORS OR COPYRIGHT
#HOLDERS BE LIABLE FOR ANY CLAIM, DAMAGES OR OTHER LIABILITY, WHETHER IN AN ACTION OF
#CONTRACT, TORT OR OTHERWISE, ARISING FROM, OUT OF OR IN CONNECTION WITH THE SOFTWARE
#OR THE USE OR OTHER DEALINGS IN THE SOFTWARE.

#python 2.7 code, requires numpy
import random
from collections import defaultdict
from numpy import median, std

def sampler(pop, size, replacement=False):
    '''a quick re-implementation of the python random sampler that
       allows for sampling with or without replacement (pythons builtin only
       allows without replacement)'''
    if replacement:
        return [random.choice(pop) for i in xrange(size)]
    else:
        return random.sample(pop, size)

def count_all(xlist, proportions=False):
    '''Count all the items in a list, return a dict
       with the item as key and counts as value'''
    out =  defaultdict(int)
    for i in xlist: out[i]+=1
    if proportions:
        out2 = {}
        tot_sz = float(sum(out.values()))
        for i in out: out2[i] = out[i] / tot_sz
        return out2
    else: return out

fams = [('fam.11', 101,145),('fam.37', 120,182),('fam.24', 62,368)]#samp sizes for
families 11, 37, and 24
geno = 'ab'#2 alleles each at 50% freq, this will maximize variance
sampler_gen = lambda size: count_all([''.join(sorted(sampler(geno,2,1))) for i in
range(size)],1)
print '(fam,s1,s2)\t     genotype\tMAD\tSD'
for fam in fams:
    ffam, c1, c2 = fam
    d = [[sampler_gen(c1), sampler_gen(c2)] for i in xrange(5000)]
    for i in ['aa', 'ab', 'bb']:
```

```
        if fam == ('fam.24', 62, 368) and i == 'ab': star='*'
        else: star = ''
        abs_dev = [abs(q[0][i]-q[1][i]) for q in d]
        print '\t'.join(map(str, [fam, i, round(median(abs_dev),5),
round(std([q[0][i]-q[1][i] for q in d], ddof=1),5), star]))
```
