# Supplementary material for: Genetic Markers for Western Corn Rootworm Resistance to Bt Toxin
Source: G3 (Bethesda). 2015 Jan 7;5(3):399–405. doi: 10.1534/g3.114.016485 (PMC4349093; doi:10.1534/g3.114.016485)
Supplement: Supporting Information [file supp_g3.114.016485_FigureS2.pdf]

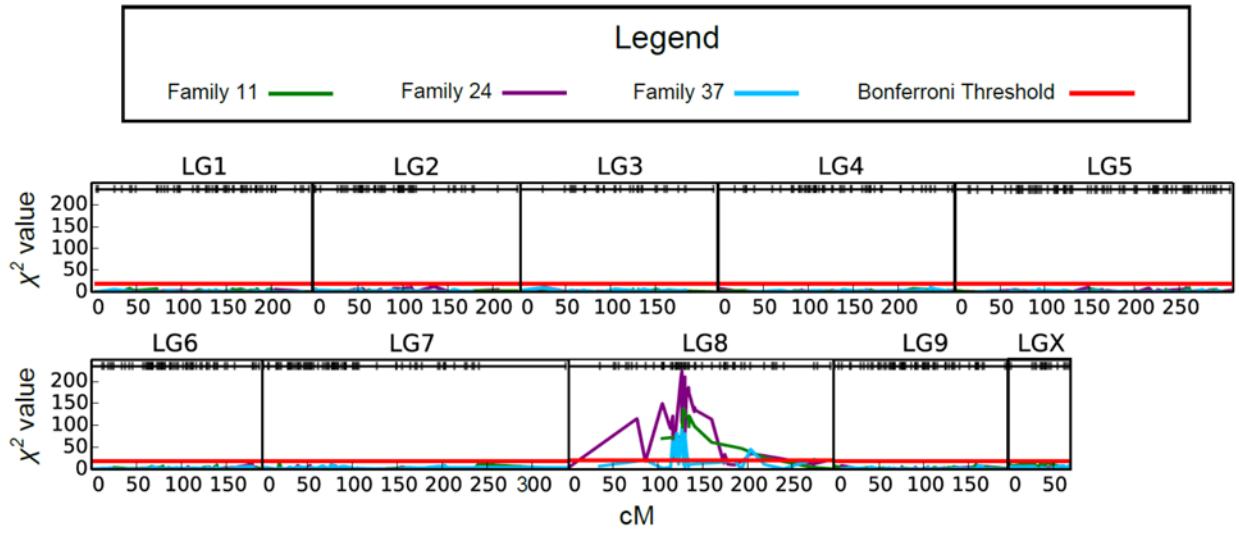

Figure S2 Difference in genotype frequencies between treatment and control F_2 populations for all 10 linkage groups as measured by the chi-squared statistic. The plot below gives a chi-squared test of independence between genotypic counts among treatment and control F_2 survivors for all three families. In total 1,497 tests were performed, each with 2 degrees of freedom, resulting in a Bonferroni significance threshold of $\chi^2 \geq 20.6$. The genotyped marker positions are given at the top of each linkage group panel.
