# Supplementary material for: Genetic Markers for Western Corn Rootworm Resistance to Bt Toxin
Source: G3 (Bethesda). 2015 Jan 7;5(3):399–405. doi: 10.1534/g3.114.016485 (PMC4349093; doi:10.1534/g3.114.016485)
Supplement: Supporting Information [file supp_g3.114.016485_FigureS3.pdf]

A.

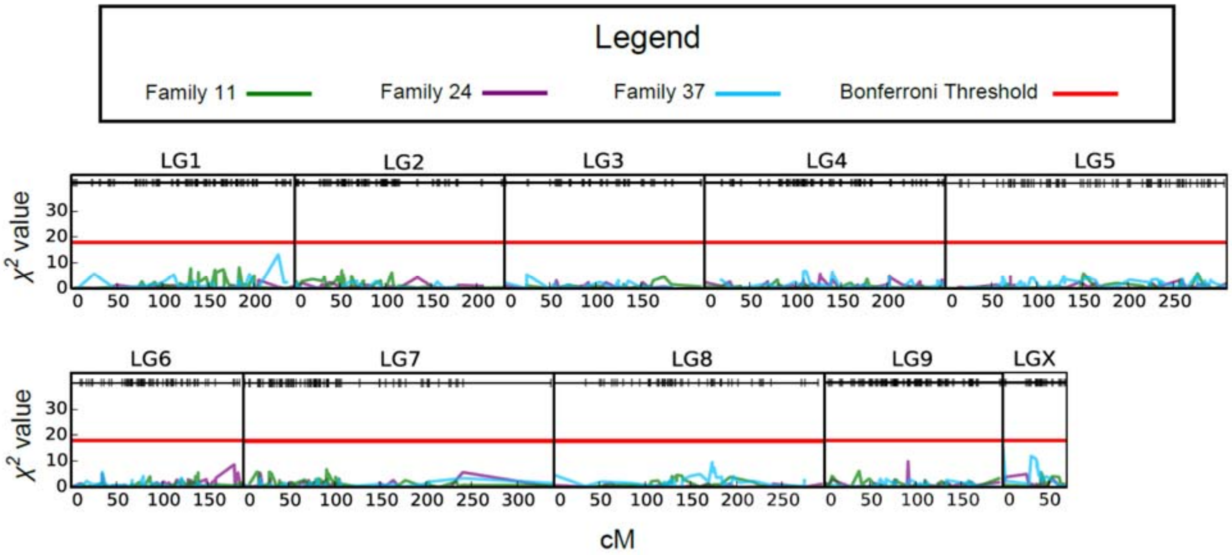

B.

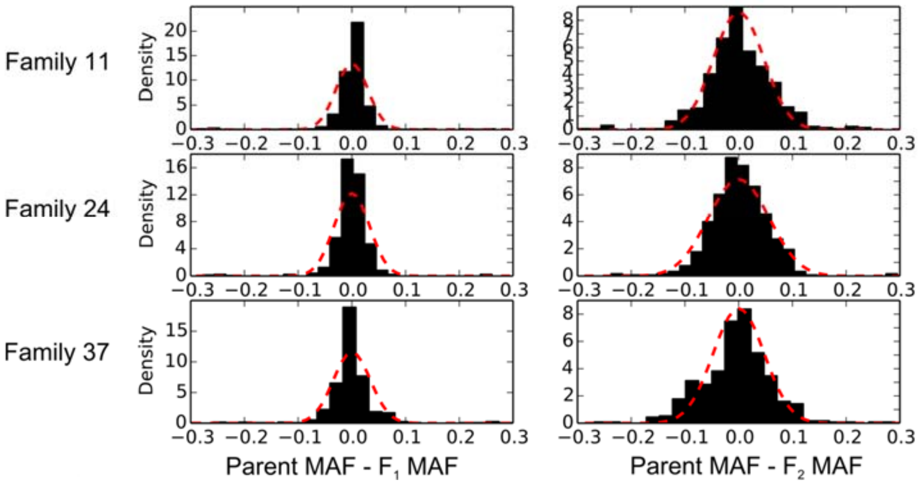

Figure S3 Tests for segregation distortion among mapping families. **(A)** The genomic context of allele frequency segregation distortion between F_1s and the control F_2s across the 10 linkage groups as measured by the chi-squared statistic. 1,713 tests were run, each with 1 degree of freedom, resulting in a Bonferroni significance threshold of $\chi^2 \geq 17.5$. No markers show significant segregation distortion at this threshold. The genotyped marker positions are given at the top of each linkage group panel.

(B) Histograms of the deviation of the parental minor allele frequency (MAF) and the frequency of that same allele in the F_1 and control F_2 generations (black bars) for each of the three families and theoretical expectations based on random genetic drift (red dashed line). Allele frequency deviations are nearly symmetric and follow a distribution consistent with the expectation from random genetic drift.
