# Supplementary material for: Genetic Markers for Western Corn Rootworm Resistance to Bt Toxin
Source: G3 (Bethesda). 2015 Jan 7;5(3):399–405. doi: 10.1534/g3.114.016485 (PMC4349093; doi:10.1534/g3.114.016485)
Supplement: Supporting Information [file supp_g3.114.016485_FigureS4.pdf]

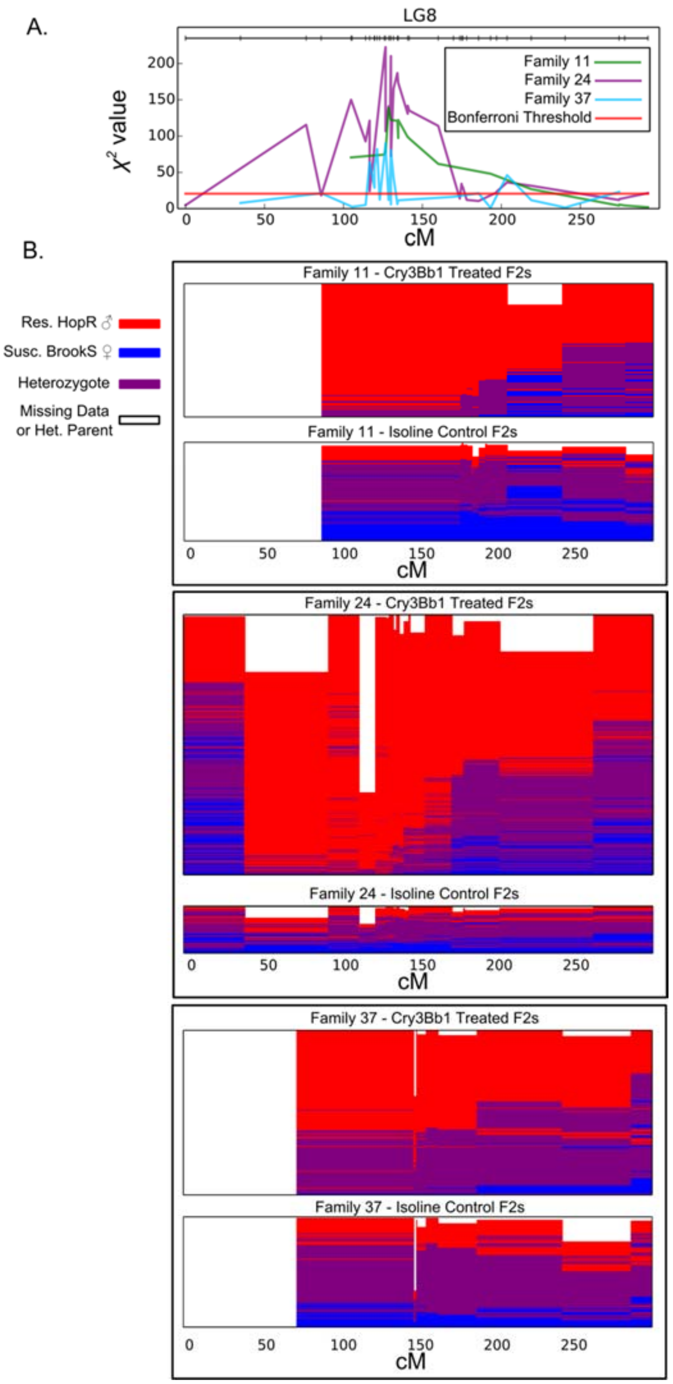

Figure S4 Resistant parent haplotype enrichment among Cry3Bb1 treated F_2s. **(A)** Map of resistance locus on LG8. **(B)** Enrichment of haplotpes contributed by Cry3Bb1 resistant parent in the resistance region on LG8 among F_2 survivors of Cry3Bb1. Each horizontal bar respresent the genotype of a single F_2 individual. Only F_2-informative markers are plotted to ensure correct haplotype phasing. Cry3Bb1 selected F_2s show enrichment for the resistant parent halpotype (red), while all control F2s segregate near the expected 1:2:1 ratio among the resistant parent (red), heterozygote (purple), and susceptible parent (blue) genotypes. Data are missing for the left portion of Family 11 and 37 because the mapping parents lacked F_2-informative markers in this region.
