# Supplementary material for: Genetic Markers for Western Corn Rootworm Resistance to Bt Toxin
Source: G3 (Bethesda). 2015 Jan 7;5(3):399–405. doi: 10.1534/g3.114.016485 (PMC4349093; doi:10.1534/g3.114.016485)
Supplement: Supporting Information [file supp_g3.114.016485_FigureS5.pdf]

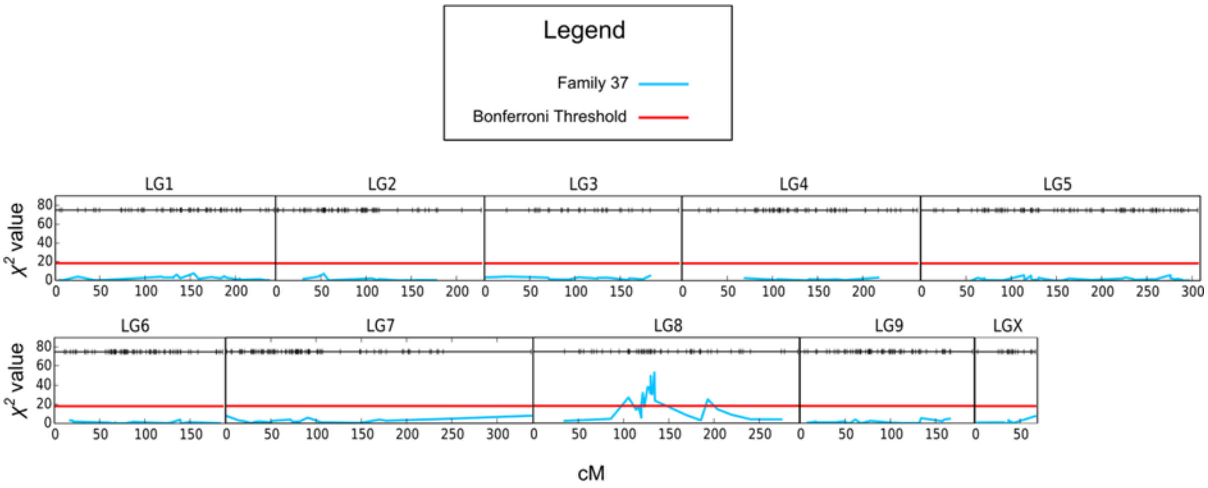

Figure S5 To search for loci unlinked to the LG8 resistance locus that may influence resistance we focused on F_2 survivors of the Cry3Bb1 treatmeant that where heterozygous in the resistance interval on LG8. Conditioning on these individuals as the treatment population, the plot below gives the difference in genotype frequencies between treatment and control F_2 populations for all 10 linkage groups as measured by the chi-squared statistic. In total, 454 tests were performed, each with 2 degrees of freedom, resulting in a Bonferroni significance threshold of $\chi^2 \geq 18.2$. Only the LG8 resistance region is significant (due to the fact that the treatment population was completely heterozygous, while the control population contained resistant parent, heterozygote, and suscpetible parent genotypes). The genotyped marker positions are given at the top of each linkage group panel.
