# Supplementary material for: Genetic Markers for Western Corn Rootworm Resistance to Bt Toxin
Source: G3 (Bethesda). 2015 Jan 7;5(3):399–405. doi: 10.1534/g3.114.016485 (PMC4349093; doi:10.1534/g3.114.016485)
Supplement: Supporting Information [file supp_g3.114.016485_FigureS6.pdf]

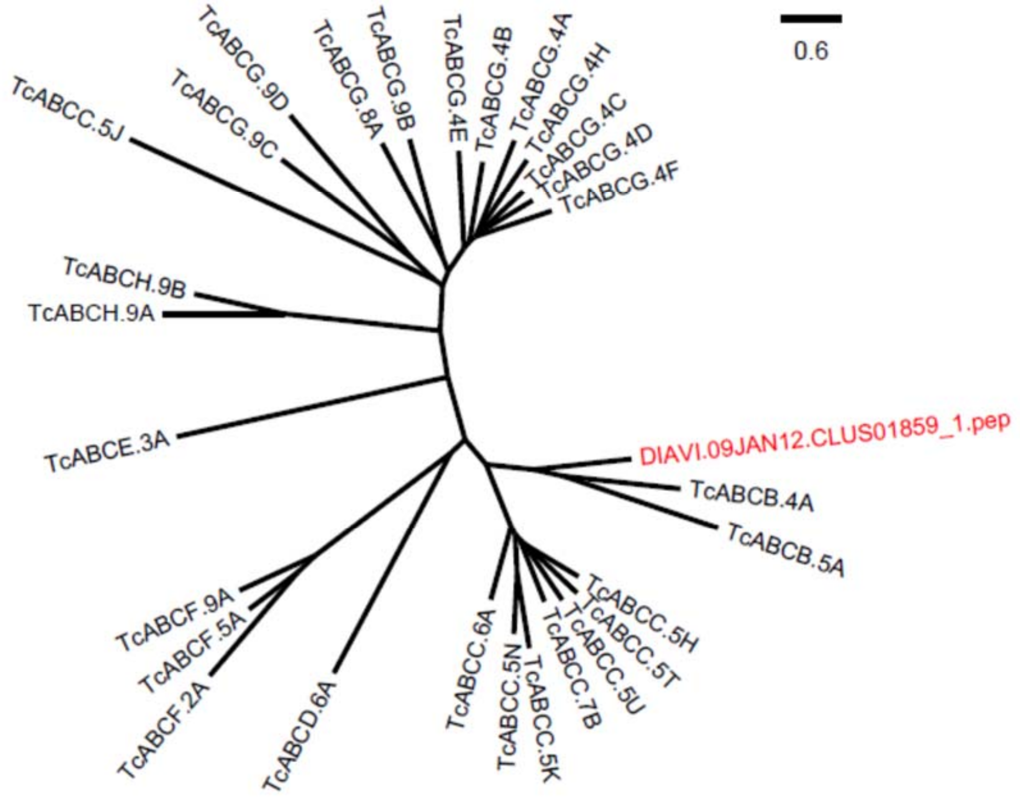

Figure S6 Neighbor joining phylogeny of ABC transporter protein sequences from *Tribolium castaneum* (black) and a WCR homolog (red). The WCR gene contains the CRW424 SNP marker linked to the LG8 resistance locus. The WCR homolog appears to be a member of the ABCB clade. The *T. castaneum* genes were annotated by Broehan *et al.*, 2013 (BMC Genomics 14:6). A substitution scale bar is given in the upper right corner.
