# Supplementary material for: Genetic Markers for Western Corn Rootworm Resistance to Bt Toxin
Source: G3 (Bethesda). 2015 Jan 7;5(3):399–405. doi: 10.1534/g3.114.016485 (PMC4349093; doi:10.1534/g3.114.016485)
Supplement: Supporting Information [file supp_g3.114.016485_FileS3.pdf]

File S3

Python simulation code

```
#Lex Flagel, Monsanto Co.
#10/29/14
#Estimating genotypic sampling error between treatment and control population using
#Monte Carlo
#
#Copyright (c) 2014 Monsanto Co.
#Permission is hereby granted, free of charge, to any person obtaining a copy of this
#software and associated documentation files (the "Software"), to deal in the Software
#without restriction, including without limitation the rights to use, copy, modify,
#merge, publish, distribute, sublicense, and/or sell copies of the Software, and to
#permit persons to whom the Software is furnished to do so, subject to the following
#conditions:
#
#The above copyright notice and this permission notice shall be included in all copies
#or substantial portions of the Software.
#
#THE SOFTWARE IS PROVIDED "AS IS", WITHOUT WARRANTY OF ANY KIND, EXPRESS OR IMPLIED,
#INCLUDING BUT NOT LIMITED TO THE WARRANTIES OF MERCHANTABILITY, FITNESS FOR A
#PARTICULAR PURPOSE AND NONINFRINGEMENT. IN NO EVENT SHALL THE AUTHORS OR COPYRIGHT
#HOLDERS BE LIABLE FOR ANY CLAIM, DAMAGES OR OTHER LIABILITY, WHETHER IN AN ACTION OF
#CONTRACT, TORT OR OTHERWISE, ARISING FROM, OUT OF OR IN CONNECTION WITH THE SOFTWARE
#OR THE USE OR OTHER DEALINGS IN THE SOFTWARE.

#python 2.7 code, requires numpy
import random
from collections import defaultdict
from numpy import median, std

def sampler(pop, size, replacement=False):
    '''a quick re-implementation of the python random sampler that
       allows for sampling with or without replacement (pythons builtin only
       allows without replacement)'''
    if replacement:
        return [random.choice(pop) for i in xrange(size)]
    else:
        return random.sample(pop, size)

def count_all(xlist, proportions=False):
    '''Count all the items in a list, return a dict
       with the item as key and counts as value'''
    out =  defaultdict(int)
    for i in xlist: out[i]+=1
    if proportions:
        out2 = {}
        tot_sz = float(sum(out.values()))
        for i in out: out2[i] = out[i] / tot_sz
        return out2
    else: return out

fams = [('fam.11', 101,145),('fam.37', 120,182),('fam.24', 62,368)]#samp sizes for
families 11, 37, and 24
geno = 'ab'#2 alleles each at 50% freq, this will maximize variance
sampler_gen = lambda size: count_all([''.join(sorted(sampler(geno,2,1))) for i in
range(size)],1)
print '(fam,s1,s2)\t     genotype\tMAD\tSD'
for fam in fams:
    ffam, c1, c2 = fam
    d = [[sampler_gen(c1), sampler_gen(c2)] for i in xrange(5000)]
    for i in ['aa', 'ab', 'bb']:
```

```
        if fam == ('fam.24', 62, 368) and i == 'ab': star='*'
        else: star = ''
        abs_dev = [abs(q[0][i]-q[1][i]) for q in d]
        print '\t'.join(map(str, [fam, i, round(median(abs_dev),5),
round(std([q[0][i]-q[1][i] for q in d], ddof=1),5), star]))
```
